# The Endothelial Tyrosine Phosphatase SHP-1 Plays an Important Role for Vascular Haemostasis in TNF****α****-Induced Inflammation *In Vivo*

**DOI:** 10.1155/2013/279781

**Published:** 2013-05-09

**Authors:** Elisabeth Koch, Joachim Pircher, Thomas Czermak, Erik Gaitzsch, Stefan Alig, Hanna Mannell, Markus Niemeyer, Florian Krötz, Markus Wörnle

**Affiliations:** ^1^Medizinische Klinik und Poliklinik IV, Innenstadt, Ludwig Maximilians Universität München, Ziemssenstr. 1, 80336 Munich, Germany; ^2^Walter Brendel Zentrum für Experimentelle Medizin, Ludwig Maximilians Universität München, Marchioninistr. 27, 81377 Munich, Germany; ^3^Frauenklinik und Poliklinik der Technischen Universität München, Technische Universität München, Ismaninger Straße 22, 81675 Munich, Germany; ^4^Invasive Kardiologie, Klinikum Starnberg, Oßwaldstr. 1, 82319 Starnberg, Germany

## Abstract

*Introduction*. Inflammation and endothelium-derived superoxides are important pathomechanisms in atherothrombotic diseases. We could previously show that the tyrosine phosphatase SHP-1 acts as a negative regulator in endothelial superoxide production. In this study we investigated the influence of SHP-1 on platelet-endothelium interaction and arterial thrombosis in TNF**α**-induced endothelial inflammation *in vivo*. *Methods*. Arteriolar thrombosis and platelet rolling *in vivo* were investigated in C57BL/6 mice using intravital microscopy in the dorsal skinfold chamber microcirculation model. *Results*. Inhibition of SHP-1 by the specific pharmacological inhibitor sodium stibogluconate did not significantly enhance platelet-endothelium interaction *in vivo* under physiological conditions but led to an augmented fraction of rolling platelets in TNF**α**-induced systemic inflammation. Accordingly, ferric-chloride-induced arteriolar thrombus formation, which was already increased by SHP-1 inhibition, was further enhanced in the setting of TNF**α**-induced inflammation. Platelet aggregation *in vitro* as well as *ex vivo* was not influenced by SHP-1-inhibition. In cultured endothelial cells, sodium stibogluconate increased TNF**α**-induced surface expression of p-selectin and von Willebrand factor. Additionally, TNF**α** increased SHP-1 activity and protein expression. *Conclusions*. The endothelial tyrosine phosphatase SHP-1 plays an important role for vascular hemostasis *in vivo,* which is crucial in TNF**α**-induced endothelial inflammation where it may serve as an autoinhibitory molecule to prevent excess inflammatory response and thrombus formation.

## 1. Introduction

Inflammatory processes play a critical role in the development of atherosclerosis and its fatal outcomes, such as myocardial infarction and ischemic stroke [1, 2]. Proinflammatory cytokines including TNF*α* and IL-1*β* contribute to progression of atherosclerotic plaque size [3, 4]. Translocation of NF-*κ*B, increased production of ROS (reactive oxygen species), or influence on eNOS expression seem to be central steps in mediating the inflammatory effects [5–9]. The roles of inflammatory cytokines and ROS in cardiovascular diseases are inseparably linked, and elevated endothelial-derived ROS activate proatherogenic signalling pathways and stimulate vascular smooth muscle cell proliferation [10, 11], scavenge endothelium-derived nitric oxide, and, thus, aggravate endothelial dysfunction [10, 12]. In addition, platelets represent an important link between inflammation, atherogenesis, and thrombosis [13–16]. Platelets can interact with the activated endothelium [17] and release proinflammatory cytokines and chemokines stored in *α*-granules such as IL-1*β* or CD40L [18], which in turn enhance endothelial inflammation and maintain the vicious circle of inflammation. It is not surprising that patients with chronic inflammatory disorders are at increased risk for cardiovascular morbidity and atherothrombotic events [19], which could not be explained by traditional cardiovascular risk factors [20–22]. We and others could previously show that systemic administration of TNF*α* mediates prothrombotic effects *in vivo* [23, 24], which at least in part are mediated via endothelial cells.

The SH2-domain containing protein tyrosine phosphatase 1 (SHP-1) is known to be a negative regulator of immune receptor signalling in lymphocytes, macrophages, and platelets, in which it is typically coactivated upon cellular stimulation to exert an autoinhibitory function [25–27]. Mice with a defect in the SHP-1 gene (“motheaten viable mice”) show increased activation of the transcription factor NF-*κ*B and consecutively elevated levels of inflammatory cytokines impacting upon the regulation of the immune response [28]. We could previously show that SHP-1 is expressed in vascular endothelial cells and negatively regulates NAD(P)H-oxidase-dependent endothelial superoxide production by inhibition of PI3 K activity and subsequent inactivation of the small GTPase Rac1; SHP-1 mRNA knockdown as well as inhibition with the pharmacological inhibitor sodium stibogluconate leads to oxidative stress in endothelial cells [29].

In this study we investigated the influence of the SHP-1 inhibitor sodium stibogluconate on platelet-endothelium interaction and arterial thrombosis in TNF*α*-induced endothelial inflammation *in vivo.*

## 2. Methods

### 2.1. Chemicals

Murine TNF*α* was from Chemicon International (USA), human TNF*α* from Reliatech (Germany), collagen was from Nycomed (Germany), and reagents for multiplate analysis were from Dynabyte (Munich, Germany); antihuman SHP-1 antibodies (C-19 and D-11) were from Santa Cruz (USA), rabbit anti-human phospho-SHP-1 (Y564) antibody was from Abcam (Cambridge, UK), anti-GAPDH antibody was from Millipore (Billerica, USA), antirabbit antibody and antimouse secondary antibodies were from Calbiochem (Darmstadt, Germany) and anti-human vWF-FITC, Anti-human p-selectin-RPE, and respective negative controls were from Abd Serotec (UK). Sodium stibogluconate and all other chemicals were from Sigma-Aldrich (Germany).

### 2.2. Animals

Animal experiments were performed in wildtype C57BL/6 mice, which were purchased from Charles River, Sulzfeld, Germany. Surgical procedures were performed under short-term anesthesia induced by a single intraperitoneal injection of Midazolam 5 mg/kg (Ratiopharm, Germany), Fentanyl 0.05 mg/kg (CuraMED Pharma, Germany), and Medetomidinehydrochloride 0.5 mg/kg (Pfizer, Germany; produced by Orion Pharma, Finland) diluted in 0.9% NaCl. After the experiments the animals were killed by injection of an overdose (2 g/kg) of sodium pentobarbital (Merial, Germany). All experiments were conducted in accordance with the German animal protection law and approved by the district government of Upper Bavaria (approval reference number AZ 55.2-1-54-2531-162-08). The investigation conforms to the Directive 2010/63/EU of the European Parliament.

### 2.3. Intravital Microscopy in the Dorsal Skinfold Chamber Microcirculatory Model

The dorsal skinfold chamber microcirculatory model was used in mice as described previously [24]. Animals with an intact microcirculation underwent carotid artery catheterization for application of drugs or injection of isolated platelets, respectively. Intravital fluorescence microscopy was performed using a modified microscope (Zeiss Axiotech Vario, Germany). Images were recorded with a digital camera (AxioCam HSm, Carl Zeiss Germany). For all *in vivo* experiments murine TNF*α* (Chemicon International, USA) was administered via a carotid artery catheter at a dose of 0.4 *μ*g/kg. This was calculated to match plasma levels of approximately 5 ng/mL, which caused effects *in vitro*. Sodium stibogluconate was infused via the carotid artery catheter in a dose calculated to match plasmal levels of approximately 10 *μ*g/mL, as it has been shown to specifically inhibit SHP-1 in this concentration [30].

### 2.4. Mouse Platelet Isolation and Staining for *In Vivo* Studies

Whole blood was drawn from anesthetized mice by cardiac puncture. To prevent blood from clotting syringes contained 10% of sodium citrate. The citrated whole blood was spun at 130 g and the obtained PRP was incubated with Carboxyfluorescein (CFDA-SE 17 *μ*mol/L, Bachem, Switzerland) in the dark for 30 minutes. Labeled platelets were then spun at 340 g and resuspended in a buffered calcium-free physiologic solution (138 mmol/L NaCl, 2.7 mmol/L KCl, 12 mmol/L NaHCO_3,_ 0.4 mmol/L NaH_2_PO_4_, 1 mmol/L MgCl_2_ × 6H_2_O, 5 mmol/L D-Glucose, and 5 mmol/L Hepes; pH 7.35). For centrifugation Iloprost (10 ng/mL, Schering, Germany) was added to prevent platelet activation. The ability of the isolated and stained platelets to aggregate was tested by platelet aggregometry.

### 2.5. Intravital Analysis of Platelet-Vessel-Wall Interaction

For intravital studies of platelet interaction with the intact vessel wall isolated and fluorescent-stained murine platelets from a donor animal were injected via a carotid artery catheter and observed in the dorsal skinfold chamber model. Movie sequences of 30 s in 4–6 vessel segments in each animal were recorded and analyzed using AxioVision Software (Carl Zeiss, Germany). Vessels with abnormal flow were excluded from analysis. From the resulting length of the platelet trace in single images, velocities of single platelets were calculated using the exposure time of each single picture. Platelet vessel wall interaction (PVWI) was expressed in frequency histograms consisting of all platelet velocities analyzed. Histograms were normalized to the maximum platelet speed within a vessel to exclude biasing influences of altered blood flow velocities between different arterioles. As a consequence a rightward shift in platelet velocity distribution within a histogram expresses less PVWI, whereas a leftwards shift signalises increased PVWI at the arteriolar wall. Platelets with less than 5% of the velocity of the fastest platelets were defined as rolling platelets.

### 2.6. Intravital Assessment of Arteriolar Thrombosis

Intravital thrombotic vessel occlusion time was assessed in arterioles of C57BL/6 mice in the dorsal skinfold chamber model. For induction of intra-arteriolar thrombosis, the ferric chloride superfusion method was used as described previously [24]. Before the experiments blood vessel flow was digitally recorded and regular blood flow was confirmed for all analyzed arterioles. To visualize vessel lumina before vessel injury 50 *μ*L of a 5% fluorescein isothiocyanate-labelled dextran solution (FITC-Dextran, MW 150,000) was infused via the carotid catheter. Injury to the vascular wall was then performed by application of 30 *μ*L of a ferric chloride solution (25 mmol/L) onto arterioles, using a standardized protocol and movies were recorded until blood flow ceased.

### 2.7. Platelet Aggregation Studies

Platelet aggregation in human platelet-rich plasma (PRP) was measured using the turbidimetric method described by Born [31]. Human PRP was obtained by centrifugation of whole citrated blood, drawn from human cubital veins at 130 g. ADP-, collagen-, or TRAP-induced platelet aggregation was measured photometrically using a 2-channel aggregometer (ChronoLog 490-2D, USA) under continuous stirring at 1000 rpm at 37°C. Written consent was obtained from platelet donors.

For mice studies blood was collected from the inferior vena cava in anesthetized mice with a syringe containing heparin. Whole blood aggregation was performed by impedance aggregometry with the Multiplate (multiple platelet function analyzer) assay (Dynabyte, Munich, Germany) according to the manufacturer's protocol. Changes in impedance expressed as aggregation amplitudes were recorded over 6 minutes in duplicates and results were expressed as mean arbitrary aggregation units (AU).

### 2.8. Cell Culture

Human umbilical vein endothelial cells (HUVECs) and porcine aortic endothelial cells (PAECs) were isolated as described previously [32] and cultured in M199 media supplemented with 10% FCS, 10% endothelial growth media (PromoCell, Germany), and 1% penicillin/streptomycin. For all cell culture experiments recombinant human TNF*α* (Reliatech, Germany) was used. The procedure was approved by a university ethic review board and the investigation conforms with the principles outlined in the Declaration of Helsinki.

### 2.9. Endothelial Surface Molecule Expression

HUVECs were grown as described and incubated with sham or recombinant TNF*α* (5 ng/mL) or sodium stibogluconate as indicated. Cells were stained using anti-p-selectin RPE and anti-vWF FITC or corresponding RPE- or FITC-labeled negative control. For measuring a FACSCanto II flow cytometer (Becton Dickinson, USA) was used. Data were analyzed using FACSDiva software (Becton Dickinson).

### 2.10. Immunoprecipitation of SHP-1 and SHP-1 Activity

PAECs were washed and lysed in radioimmunoprecipitation (RIPA) buffer and protein content determined as described elsewhere [33]. From aliquots of 300 *μ*g protein immunoprecipitations of SHP-1 were performed using a polyclonal primary anti-SHP-1 mouse antibody (C-19), MACS Protein G MicroBeads, and MACS separation columns from Miltenyi Biotec (Bergisch Gladbach, Germany) according to the manufacturer's protocol. Equal amounts of SHP-1 were then equilibrated in phosphatase assay buffer (Hepes 20 mmol/L, sodium chloride 100 mmol/L, magnesium chloride 5 mmol/L, manganese chloride 5 mmol/L, pH 6.5). After addition of 10 mmol/L of the chromogenic substrate p-nitrophenylphosphate and incubation for 1 h (37°C) solutions were transferred to multiwell plates, and extinction was measured at 405 nm (SpectraFluor, Tecan, Germany).

### 2.11. Western Blot

Protein lysates from PAEC or HUVEC (whole cell lysate) were prepared and protein content quantified as described elsewhere. Lysates were subjected to Western blot analysis as previously described [33] and SHP-1 was detected using a polyclonal rabbit anti-SHP-1 antibody (C-19). To measure SHP-1 activity phosphorylation of SHP-1 on Y564 was detected using a rabbit anti-phoshpho-Y564-SHP-1 antibody (Abcam), as phosphorylation at this site has been described to correlate with the phosphatase activity [34]. GAPDH was used as loading control.

### 2.12. Statistical Analysis

SigmaStat Software was used to calculate statistical differences. Data were analyzed using Student's *t*-test or ANOVA for normally distributed variables or the Mann-Whitney Rank Sum Test or ANOVA on ranks, when normal distribution was not given. Data are expressed as means ± SEM. Differences were considered significant when the error probability level was *P* < 0.05.

## 3. Results

### 3.1. Platelet-Endothelium Interaction *In Vivo*

We assessed transient interaction of injected labelled platelets to the vessel wall *in vivo* by intravital microscopy of vessels in the dorsal skinfold chamber. Sodium stibogluconate was used in a concentration of 10 *μ*g/mL (11 *μ*M), for which it has been shown to exhibit the by far highest affinity for SHP-1, whereas 10-fold higher concentrations were required to inhibit other tyrosine phosphatases such as SHP-2 and PTP1B [30].

**Figure 1:**
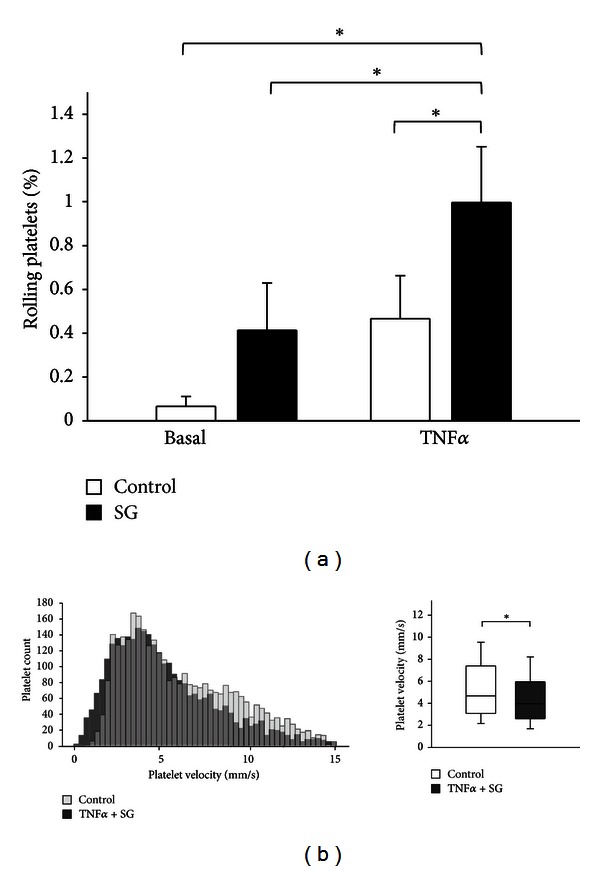
Inhibition of SHP-1 leads to increased platelet rolling *in vivo* in TNF*α*-induced systemic inflammation. (a) *In vivo* the amount of rolling platelets (defined as platelets with a velocity of less than 5% of maximal velocity) as analyzed by intravital microscopy in the dorsal skinfold chamber was slightly enhanced *in vivo* after treatment with the SHP-1 inhibitor sodium stibogluconate (10 *μ*g/mL, 30 min) under basal conditions but significantly increased in TNF*α*-induced inflammation (TNF*α* 5 ng/mL, 4 h). (b) Inhibition of SHP-1 by sodium stibogluconate in TNF*α*-induced inflammation resulted in a leftward shift in platelet velocity distribution pattern (i.e., towards lower platelet velocities) in the frequency histogram, indicating increased transient platelet interaction with the endothelium. The histograms display all platelet velocities from 5 different animals per group. SG: sodium stibogluconate. *Significantly different at *P* < 0.05 (*n* = 2900 − 3300 platelets per group from 5 different animals).

Systemic treatment with sodium stibogluconate (10 *μ*g/mL, 30 min) did slightly enhance the amount of rolling platelets under physiological conditions (0.4 ± 0.2% of all analyzed injected platelets versus 0.1 ± 0.1% without SHP-1 inhibition; *P* = 0.12, *n* = 5) but significantly elevated rolling platelets in acute systemic inflammation induced by TNF*α* treatment (5 ng/mL, 4 hours; 1.0 ± 0.3% rolling platelets versus 0.4 ± 0.2% without SHP-1 inhibtion, *P* < 0.05, *n* = 5, Figure 1(a)). Increased transient platelet-endothelium interaction after SHP-1 inhibition by sodium stibogluconate in TNF*α*-induced inflammation is also indicated by a leftward shift in the platelet flow velocity histogram (velocities from 2900–3300 analyzed platelets from 5 different animals in each group, Figure 1(b)). The maximum platelet velocities in the analyzed vessels as an approximate measure of flow velocity were not significantly different between the treatment groups.

### 3.2. Arteriolar Thrombus Formation *In Vivo*

**Figure 2:**
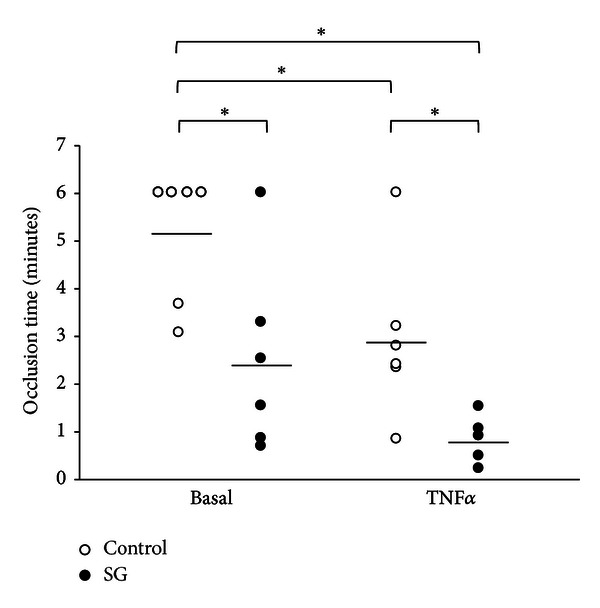
Inhibition of SHP-1 leads to accelerated arteriolar thrombus formation *in vivo*. Time to thrombotic arteriolar vessel occlusion *in vivo*, as measured in the ferric chloride superfusion model in the dorsal skinfold chamber in mice, was significantly accelerated when animals were treated with the SHP-1 inhibitor sodium stibogluconate (10 *μ*g/mL, 30 min) under basal conditions. TNF*α*-induced inflammation itself (TNF*α* 5 ng/mL, 4 h) led to accelerated thrombus formation, but the effect was further increased by SHP-1 inhibition. SG: sodium stibogluconate. *Significantly different at *P* < 0.05 (*n* = 5–6).

To analyze the effect of SHP-1 inhibtion on thrombus formation *in vivo*, we assessed the time to thrombotic arteriolar vessel occlusion following injury by ferric chloride superfusion to the vascular wall. The time to complete thrombotic vessel occlusion upon injury was significantly accelerated from 307 ± 34 s in control animals to 149 ± 49 s in animals treated with the SHP-1 inhibitor sodium stibogluconate 10 *μ*g/mL for 30 minutes (*n* = 6, *P* < 0.05 versus control animals). Vessel occlusion time was further accelerated by SHP-1 inhibition in TNF*α*-induced (5 ng/mL, 4 hours) inflammation (50 ± 14 s versus 176 ± 42 s without SHP-1 inhibtion; *n* = 5; *P* < 0.05, Figure 2).

### 3.3. Influence of SHP-1 Inhibtion on Endothelial P-Selectin and vWF Surface Expression

**Figure 3:**
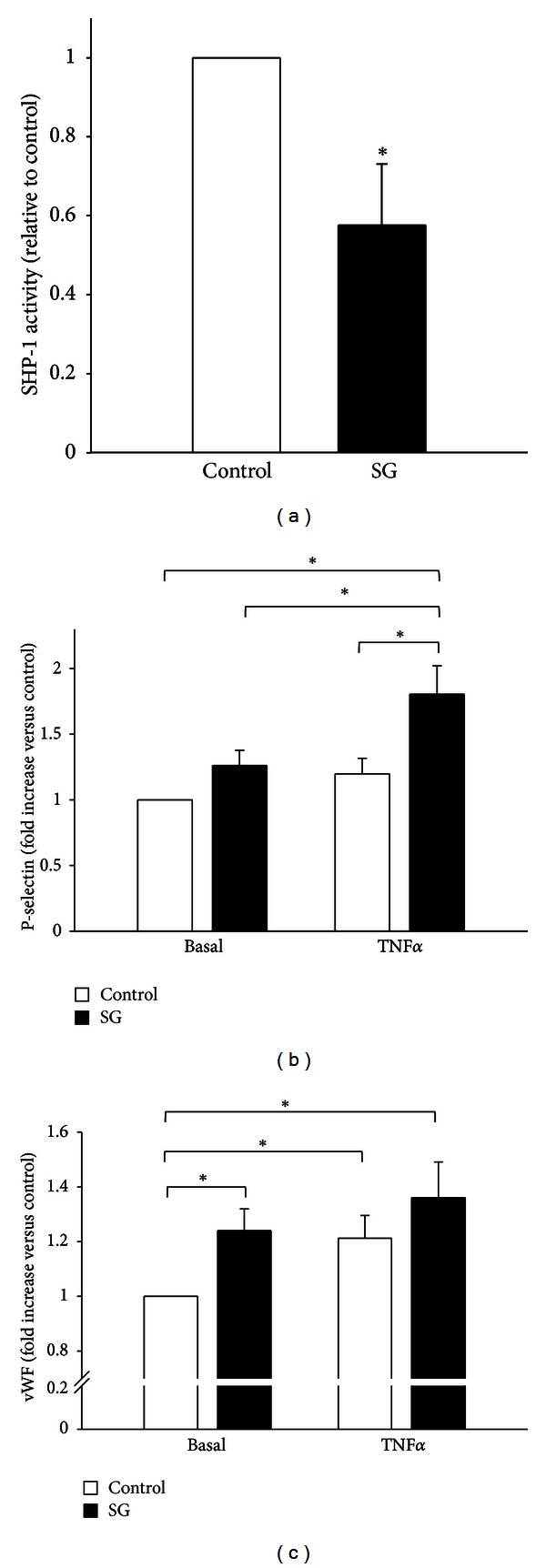
Inhibition of SHP-1 increases TNF*α*-induced upregulation of p-selectin and vWF in endothelial cells *in vitro*. (a) Incubation of endothelial cells with the pharmacological SHP-1 inhibitor sodium stibogluconate effectively reduces SHP-1 activity as measured by pNPP-dephosphorylation assay. (b) Endothelial p-selectin was not changed by sodium stibogluconate under basal conditions but, significantly upregulated in TNF*α*-induced endothelial inflammation. (c) vWF was already upregulated by sodium stibogluconate under basal conditions to a similar level as observed in TNF*α*-induced endothelial inflammation. SG: sodium stibogluconate. *Significantly different at *P* < 0.05 (HUVEC, *n* = 9–12).

To better define the relative contribution of the endothelium to the prothrombotic effects exerted by SHP-1 inhibtion in TNF*α*-induced inflammation we analyzed adhesion molecules relevant to platelet-endothelium interaction *in vitro* in primary human endothelial cells (HUVECs) by flow cytometry. First we tested whether sodium stibogluconate inhibits SHP-1 in endothelial cells and measured SHP-1 activity by the enzymatic pNNP-dephosphorylation assay. Treatment with sodium stibogluconate (10 *μ*g/mL, 30 min) reduced SHP-1 activity in HUVEC to 58 ± 15% of baseline activity (*n* = 7–8, *P* < 0.05, Figure 3(a)).

P-selectin was not significantly upregulated by the SHP-1 inhibitor sodium stibogluconate (10 *μ*g/mL, 30 min) under basal conditions (120 ± 11% of baseline, *n* = 9–12; *P* = 0.06) but was significantly increased by SHP-1 inhibition in TNF*α*-induced (5 ng/mL, 4 hours) inflammation (180 ± 22% of baseline and 126 ± 11% of baseline without SHP-1 inhibtion, *n* = 12, *P* < 0.05, Figure 3(b)).

Next we measured vWF on the surface membrane of HUVEC, which was upregulated by the SHP-1 inhibitor sodium stibogluconate (10 *μ*g/mL, 30 min) already under basal conditions (124 ± 8% of baseline, *n* = 20, *P* < 0.05) and showed a tendency to be further increased by SHP-1 inhibtion in TNF*α*-induced (5 ng/mL, 4 hours) inflammation (136 ± 13% of baseline versus 121 ± 8% of baseline without SHP-1 inhibtion, *n* = 9, n.s.; both *P* < 0.05 versus control, Figure 3(c)).

### 3.4. Platelet Aggregation

**Figure 4:**
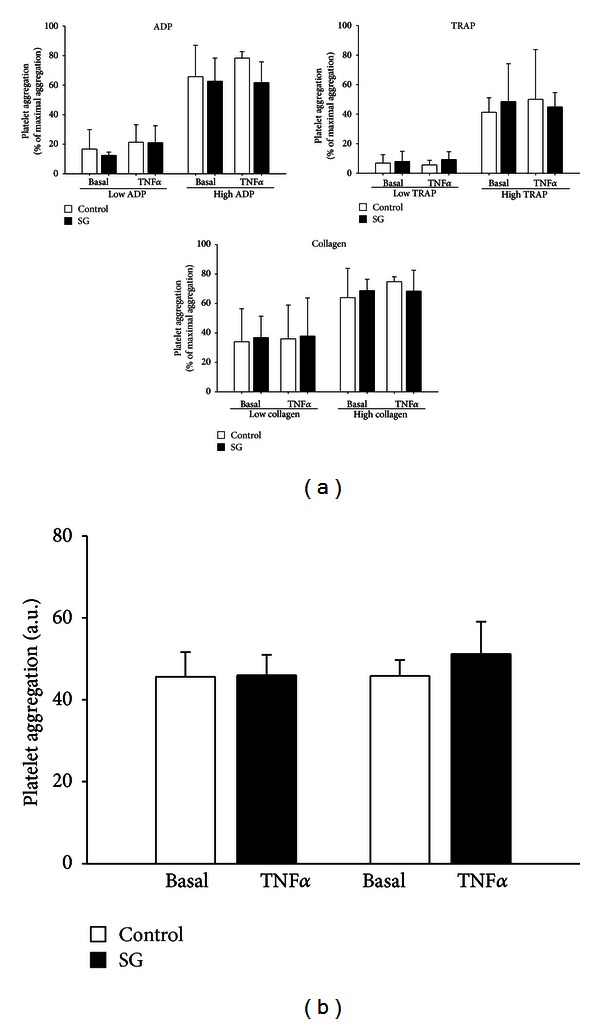
Inhibtion SHP-1 does not affect platelet aggregation *in vitro* and *ex vivo*. (a) In platelet-rich plasma (PRP) from healthy human volunteers SHP-1 inhibition by sodium stibogluconate did not increase platelet aggregation neither under basal conditions nor after pretreatment with TNF*α* (5 ng/mL, 4 h) upon stimulation with the platelet agonists ADP, collagen, or thrombin-receptor activating peptide (TRAP) in different concentrations (low ADP: 0.5–1 *μ*M; high ADP: 10 *μ*M; low collagen: 0.5–4 *μ*g/mL; high collagen: 10 *μ*g/mL; low TRAP: 1–1.5 *μ*M; high TRAP: 10–14 *μ*M; *n* = 9–12, each). (b) Treatment of animals with the SHP-1 inhibitor sodium stibogluconate (10 *μ*g/mL, 30 min) did not affect ADP-induced (6.5 *μ*M) platelet aggregation *ex vivo*, neither under basal conditions nor in TNF*α*-induced inflammation (TNF*α* 5 ng/mL, 4 h, *n* = 6 animals per group). SG: sodium stibogluconate, AU: arbitrary unite.

To test for direct effects of the SHP-1 inhibitor sodium stibogluconate and TNF*α* on platelets we assessed platelet aggregation *in vitro* in human PRP using light transmission aggregometry (Born's method). Incubation of PRP with human TNF*α* 5 ng/mL for 4 hours and additional inhibition of SHP-1 (sodium stibogluconate 10 *μ*g/mL for 30 min) did not affect platelet aggregation, upon stimulation neither with low nor with high doses of the platelet agonists ADP, collagen, and thrombin-receptor-activating peptide (TRAP; *n* = 9–12, each, Figure 4(a)).

To test effects of systemic SHP-1 inhibition on platelets aggregation was measured in whole blood of mice after oral treatment with sodium stibogluconate, which did not change ADP-induced (ADP 6.5 *μ*M) platelet aggregation compared to control treated animals, neither under basal conditions nor in TNF*α*-induced inflammation (*n* = 6 animals per group, Figure 4(b)).

### 3.5. Influence of TNF*α* on SHP Activity and Expression

To investigate whether TNF*α* affects SHP-1 phosphatase activity and protein expression we measured SHP-1 phosphatase activity by pNPP-dephosphorylation assay and by detection of SHP-1 phosphorylation at tyrosine 564 by Western blot in primary endothelial cells (PAEC and HUVEC, resp.).

**Figure 5:**
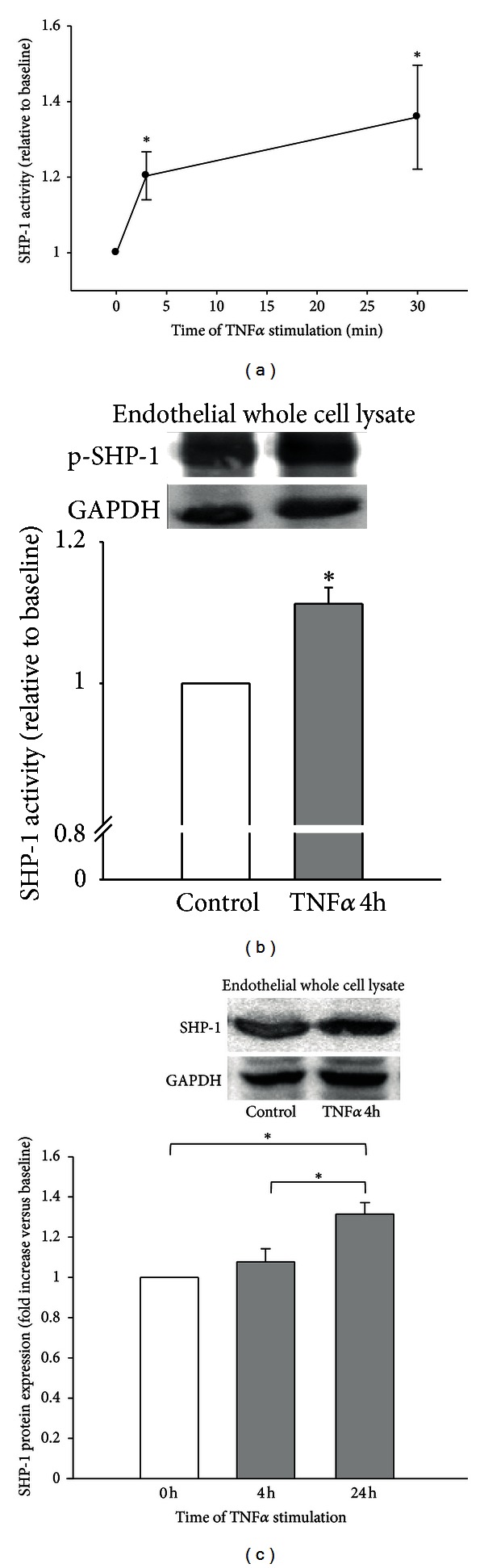
TNF*α* increases SHP-1 activity and protein expression. (a) TNF*α* (5 ng/mL) treatment of endothelial cells increased SHP-1 activity already within minutes of stimulation as assessed by pNPP-dephosphorylation assay in PAEC (*n* = 6–8). (b) This could also be observed in HUVEC after TNF*α* stimulation (5 ng/mL, 4 h) as detected by phosphorylation of SHP-1 at Y564. (c) SHP-1 protein expression was not changed within the first hours of stimulation but significantly increased after 24 hours of stimulation. *Significantly different versus baseline or as indicated at *P* < 0.05 (*n* = 6–8).

TNF*α* treatment (5 ng/mL) of PAEC caused a rapid increase of SHP-1 phosphatase activity to 120 ± 6% of baseline after 3 minutes and 136 ± 14% of baseline after 30 minutes (*n* = 6; *P* < 0.05 versus control, Figure 5(a)) in PAEC. Similarly, SHP-1 activity was increased by TNF*α* treatment (5 ng/mL for 4 h) in HUVEC (112 ± 2% of baseline, *n* = 4; *P* < 0.05; Figure 5(b)).

SHP-1 protein expression was not changed by TNF*α* (5 ng/mL) after 4 hours of stimulation but was significantly elevated after 24 hours (132 ± 6% of baseline, *n* = 8, *P* < 0.05; Figure 5(c)).

## 4. Discussion

In this study we show that inhibition of the tyrosine phosphatase SHP-1 results in increased platelet-endothelium interaction and accelerated arteriolar thrombus formation *in vivo*, possibly by upregulation of adhesion molecules on endothelial cells. These effects are further potentiated in the setting of TNF*α*-induced inflammation. Since TNF*α* augments the activity of SHP-1, the tyrosine phosphatase may serve as an autoinhibitory feedback mechanism, which is important to prevent excess inflammatory responses and thrombus formation.

In our *in vivo* studies inhibition of treatment with the SHP-1 inhibitor sodium stibogluconate led to a slightly greater amount of rolling platelets as compared to control treated animals under physiological conditions. However, this effect was significantly increased when SHP-1 was inhibited in an acute inflammatory setting caused by systemic TNF*α* treatment, resulting in significantly elevated transient platelet-endothelium interaction. When the endothelium is activated, similar to leukocytes, platelets can roll on the endothelium [17]. Under shear stress p-selectin and its counterreceptors PSGL-1 or GPIb and vWF are mediating platelet interaction with the endothelium and trigger the release of proinflammatory cytokines and chemokines stored in *α*-granules such as p-selectin or the chemokine-like factors IL-1*β* and CD40L [18] which in turn can activate the endothelium. Indeed, *in vitro* in cultured endothelial cells inhibtion of SHP-1 by sodium stibogluconate led to rapid upregulation of p-selectin in a TNF*α*-induced inflammatory condition, whereas a significant upregulation of vWF was observed already under basal conditions, which fits very well in with our *in vivo* results. These adhesion molecules have been described to be upregulated by ROS not only via redox-sensitive transcription factors such as NF-*κ*B [12] but also by rapid release from Weibel-Palade bodies by a posttranslational cell signaling response mediated by not only classical NADPH oxidase but also by xanthine oxidase [35]. We have previously identified SHP-1 as a negative regulator of endothelial NADPH-oxidase dependent superoxides already under basal conditions [29], which highly suggests a role for ROS to be involved in mediating these effects.

Moreover inhibition of SHP-1 already under basal condition (i.e., without prior stimulation with TNF*α*) led to a significantly accelerated thrombotic vessel occlusion time *in vivo* in dorsal skin arterioles upon ferric chloride injury. This effect was even more pronounced in TNF*α*-induced inflammation. We have previously shown that TNF*α* can increase arteriolar thrombosis *in vivo* by several mechanisms on the transcriptional level including upregulation of tissue factor, PAI-1, and downregulation of thrombomodulin [24]. Inhibtion of SHP-1 activity for only a short time again suggests that rapid upregulation of endothelial vWF and p-selectin, probably involving generation of ROS, is responsible for the potentiation of TNF*α*-induced prothrombotic effects. Elevated expression of vWF on endothelial cells has been shown to contribute to microvascular thrombosis *in vivo* [36] and oxidative stress is well described to induce prothrombotic changes [12].

It should be mentioned, though, that from the thrombus formation experiment direct effects of SHP-1 inhibition in platelets cannot be excluded. This is of special interest especially in the light of a previous study where platelets isolated from “motheaten viable mice”, which have a defect in the SHP-1 gene, were hyporesponsive to GPVI stimulation suggesting a physiological role for SHP-1 in lowering the threshold for activation by GPVI [27]. In addition, a role for SHP-1 in the regulation of platelet eNOS has been reported [37]. To elucidate whether the observed prothrombotic effects *in vivo* could be due to direct effects of SHP-1 inhibition by sodium stibogluconate in platelets we performed *in vitro* (human PRP) and *ex vivo* (mouse blood) aggregation studies. Interestingly, in the concentration where striking effects on endothelial cells were detected, no changes in platelet aggregation *in vitro*, neither by sodium stibogluconate itself nor after prior stimulation with TNF*α*, compared to untreated platelets in response to several platelet-activating stimuli such as collagen, ADP and TRAP could be observed. Similarly, *ex vivo* platelet aggregation in mouse whole blood after treatment with sodium stibogluconate, which caused accelerated thrombus formation *in vivo*, was not affected, neither under basal conditions nor in TNF*α*-induced inflammation. Therefore we conclude that the effects we observed in the *in vivo* experiments are due to endothelial mechanisms rather than direct effects on platelets.

The potentiation of TNF*α*-induced proinflammatory or prothrombotic effects by inhibition of SHP-1 activity in endothelial cells could be explained by an autoinhibitory feedback mechanism of the phosphatase. Autoinhibitory functions for SHP-1 have been described in other cell types, such as lymphocytes, macrophages, and platelets, where the phosphatase is coactivated and serves as a negative regulator of immune receptor signaling [25–27]. In this sense we could previously show that inhibition of SHP-1 in endothelial cells leads to increased VEGF-dependent superoxide formation [29]. In bovine aortic endothelial cells, TNF*α* has been shown to activate SHP-1 and inhibit growth factor-mediated cell proliferation through this mechanism [38]. Accordingly, Sugano et al. showed that TNF-alpha modulates angiogenic processes probably by employing SHP-1, as SHP-1 inhibition increased endothelial cell growth. Interestingly, this was shown to be caused by an impairment of the ability of TNF*α* to block the tyrosine phosphorylation of VEGFR2 induced by VEGF, strongly suggesting that SHP-1 exhibits a negative feedback regulation in TNF*α* signaling [39]. Several proteins are tyrosine phosphorylated downstream of TNF-receptor activation, involving src- and jak-family kinases [40], which in part have been shown to be regulated by SHP-1 [41]. In endothelial cells TNF*α* caused SHP-1 phosphatase activation starting already within minutes after stimulation. As SHP-1 inhibition led to prothrombotic changes, which are especially pronounced in TNF*α* induced inflammation, we propose that activated SHP-1 plays an important autoinhibitory role in acute TNF*α*-signaling. Interestingly, protein levels of SHP-1 in endothelial cells were not significantly elevated after 4 hours of TNF*α* stimulation but increased after 24 hours, suggesting also a function for SHP-1 in chronic inflammatory processes.

Considering the crucial role of inflammatory cytokines and oxidative stress in the pathophysiology of cardiovascular diseases these factors are interesting targets for pharmacological approaches. Numerous studies targeting inflammatory cytokines by antibodies or ROS by antioxidants have been performed, which, however, led to conflicting results regarding a clinical benefit and have not become established as standard therapy in cardiovascular diseases [42–44]. The understanding of the pathophysiological processes leading to cardiovascular diseases is therefore fundamental to develop new therapeutic strategies. SHP-1 has already been tested as a therapeutic target in ischemic conditions such as stroke or myocardial infarction, where inhibition of its activity resulted in a reduction of infarct sizes [45–47]. Inhibition of another tyrosine phosphatase, namely PTP1B, resulted in improvement of peripheral endothelial dysfunction in heart failure [48]. The cellular effects and functions of the numerous different cytosolic and membrane-bound tyrosine phosphatases are multiple and many times antagonistic. Thus, preservation of tyrosine phosphorylation as a therapeutic principle for vascular medicine, especially using tyrosine phosphatase inhibitors, must be viewed in a very differentiated manner and evaluated with caution.

## 5. Conclusion

Our findings indicate that the endothelial tyrosine phosphatase SHP-1 plays an important role for vascular hemostasis, which is of great relevance in TNF*α*-induced endothelial inflammation where it may serve as an autoinhibitory molecule to prevent excess inflammatory response. Eventually decreased SHP-1 activity in an inflammatory setting increases platelet-endothelium interaction and accelerates arteriolar thrombus formation *in vivo*.

## Abbreviations

ADP: Adenosine diphosphate
eNOS: endothelial nitric oxide synthase
HUVECs: Human umbilical vein endothelial cells
IL-1*β*: Interleukin-1-beta
NF-*κ*B: Nuclear-factor-Kappa-B
PAECs: Porcine aortic endothelial cells
PAI-1: plasminogen-activator-inhibitor 1
PI3 K: Phosphatidylinositide 3-kinase
pNPP: p-Nitrophenyl phosphate
PRP: Platelet-rich plasma
PSGL-1: P-selectin glycoprotein ligand-1
ROS: Reactive oxygen species
SHP-1: SH2-domain containing protein tyrosine phosphatase 1
TNF*α*: Tumor necrosis factor alpha
TRAP: Thrombin-receptor-activatingpeptide
VEGF: Vascular endothelial growth factor
VEGFR: Vascular endothelial growth factor receptor
vWF: vonWillebrandfactor.
